# Single-Cell Transcriptomic Profiling of Longissimus Dorsi and Biceps Femoris Muscles in Kazakh Horses Reveals Cellular Heterogeneity and Myogenic Regulation

**DOI:** 10.3390/ani15192778

**Published:** 2025-09-23

**Authors:** Jianwen Wang, Zexu Li, Luling Li, Ran Wang, Shikun Ma, Yi Su, Dehaxi Shan, Qiuping Huang

**Affiliations:** 1 College of Animal Science, Xinjiang Agricultural University, Urumqi 830052, China; 2 Xinjiang Key Laboratory of Equine Breeding and Exercise Physiology, Urumqi 830052, China

**Keywords:** biceps femoris muscle, gene expression, Kazakh horses, longissimus dorsi muscle, muscle specialization, single-cell RNA sequencing

## Abstract

Kazakh horses are renowned for their endurance and adaptability, with distinct muscle groups such as the longissimus dorsi (LD) and biceps femoris (BF) muscles serving specialized functions. However, the molecular mechanisms underlying the functional specialization of these muscles in Kazakh horses remain poorly understood. This study aims to address this gap by utilizing single-cell RNA sequencing (scRNA-seq) to investigate the transcriptomic differences between these muscle groups, with a focus on understanding their molecular adaptations. Our analysis revealed that the BF muscle, specialized for explosive movements, exhibited upregulation of genes associated with anaerobic metabolism, muscle contraction, and oxidative stress response, reflecting its reliance on glycolysis for sustained energy production. In contrast, the LD muscle, primarily responsible for postural support and endurance, showed a metabolic shift toward lipid utilization and energy production. Differential gene expression analysis also revealed distinct enrichment in biological pathways, with LD cells being enriched in pathways related to muscle contraction and calcium signaling, while BF cells were enriched in energy metabolism pathways. These findings provide valuable insights into the molecular adaptations of Kazakh horses’ muscle tissues, highlighting the functional specialization of LD and BF muscles and offering a foundation for future research on improving muscle performance and breeding programs in equines.

## Simple Summary

Kazakh horses are famous for thriving in harsh climates and for outstanding endurance. They rely on different muscles for different jobs: the longissimus dorsi (LD) along the back supports posture and stamina, while the biceps femoris (BF) in the hindlimb powers fast, strong movements. Using single-cell RNA sequencing, we mapped the cell types in LD and BF and asked how they work at the molecular level. We found that LD is tuned for long-lasting activity—shifting toward lipid (fat) metabolism and energy production. By contrast, BF appears primed for quick bursts—showing higher expression of genes for muscle contraction and glycolytic (sugar-based) energy, together with signals of oxidative stress response. These results reveal clear, cell-level specializations of two key equine muscles and suggest candidate markers (e.g., MYL1, LDHA) that may inform training and selective breeding for performance and health in Kazakh horses. As this study analyzed three male horses, future work with larger, diverse cohorts and targeted validation (e.g., qPCR) will further strengthen these insights.

## 1. Introduction

Skeletal muscle is a critical tissue in horses, representing over 40% of the total body mass, and plays a key role in locomotion, posture maintenance, and metabolic regulation. Among the various muscle groups, the longissimus dorsi (LD) and biceps femoris (BF) muscles are two prominent muscles that differ significantly in anatomical position and functional specialization [1,2]. The LD muscle, running along the vertebral column, is primarily involved in posture maintenance and back extension, while the BF muscle, located in the hindlimb, is crucial for forward propulsion and limb movement during locomotion. These differences reflect underlying variations in muscle fiber composition, mitochondrial activity, and mechanical loading, all of which contribute to differences in muscle performance and meat quality.

The importance of horses in agricultural and economic systems cannot be overstated. The horse industry is one of the most significant and effective sectors in the global economy, contributing substantially to wealth and income generation. It is also one of the most job-generating industries, encompassing the production, breeding, and maintenance of horses, as well as their training for competitions and related services, and the manufacturing of riding equipment. As such, improving breeding programs, enhancing genetic traits, and studying horses from various aspects is crucial. As previous studies have shown, optimizing breeding programs and enhancing genetic traits have broad applications in improving equine performance, health, and productivity [3,4].

Although studies on skeletal muscle biology in equines are emerging, particularly using transcriptomic approaches, comprehensive single-cell RNA sequencing (scRNA-seq) studies comparing different muscle types in horses are still lacking. While similar studies have been conducted in other species such as mice and humans to understand muscle specialization and gene expression in various muscle types [3,4,5], equine data remain scarce. This study represents the first scRNA-seq comparison of equine muscle types, specifically the LD and BF muscles in Kazakh horses, addressing a significant gap in equine muscle biology. By providing insights into the molecular mechanisms underlying functional specialization of these muscles, our research holds unique value in the context of horse breeding and performance optimization. The identification of key differentially expressed genes (DEGs) such as MYL1 and LDHA, which have been well-studied in human and mouse skeletal muscle, provides a comparative foundation for future studies [6,7]. These DEGs are involved in muscle contraction and energy metabolism pathways, and their conserved roles across species highlight their potential to serve as markers for improving muscle performance and health in horses, as well as for enhancing breeding programs targeting muscle function in equines.

The Kazakh horse (*Equus caballus*) is a breed native to both China and Kazakhstan, with particular significance in China, where it is the second most numerous horse breed [8]. This breed is valued for its endurance, resistance to harsh environments, and versatility in both riding and meat production. Kazakh horses are known for their strong tolerance to coarse forage, excellent cold resistance, suitability for mountain riding, high milk yield, high slaughter rate, and good meat quality. While research on the genome and phenotypic traits of Kazakh horses has begun, there is still limited understanding of the molecular mechanisms driving skeletal muscle development and differentiation in this breed [9]. In particular, the cellular composition and regulatory pathways that govern the functional differences between muscle groups like the longissimus dorsi (LD) and biceps femoris (BF) muscles remain poorly characterized.

Traditional transcriptomic approaches, such as bulk RNA sequencing, have provided insights into gene expression in muscle tissues [10]. However, these methods average signals across a heterogeneous cell population, thus obscuring the contributions of distinct cell types. Skeletal muscle is a complex tissue consisting of various cell types, including satellite cells, myoblasts, fibro-adipogenic progenitors, endothelial cells, and immune cells. These cells interact dynamically to support muscle growth, repair, and specialization. To unravel this complexity and uncover the regulatory networks governing muscle differentiation, a higher-resolution approach is required.

Single-cell RNA sequencing (scRNA-seq) has recently emerged as a powerful tool to resolve cellular heterogeneity and to reconstruct developmental trajectories in tissues such as skeletal muscle [11]. This technique has been successfully applied to investigate muscle biology in several species, including mice, pigs, and cattle, offering new insights into muscle regeneration, stem cell biology, and tissue specialization [3]. However, single-cell transcriptomic data from equine skeletal muscle remain scarce, and no studies to date have systematically compared different muscle types at single-cell resolution in horses.

In this study, we employed scRNA-seq to analyze the LD and BF muscles of three healthy 3-year-old male Kazakh horses. The goals of this study were to: (1) map the cellular landscape of each muscle type, (2) characterize the differences in cell-type composition and gene expression profiles between the two muscle types, and (3) identify key regulatory pathways involved in muscle development and specialization. This work aims to provide a comprehensive cellular atlas of skeletal muscle in horses, which will enhance our understanding of muscle biology and inform future research on muscle-related traits, performance, and breeding strategies.

## 2. Materials and Methods

### 2.1. Animal Sample Collection

**Figure 1:**
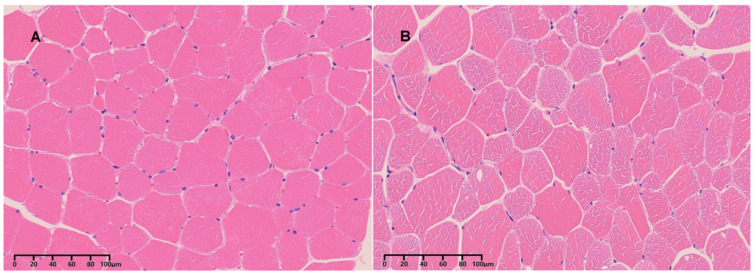
Muscle fiber histological characteristics. (**A**) LD; (**B**) BF.

Three healthy 3-year-old male Kazakh horses were selected from a livestock farm in Xinjiang, China. These horses were chosen based on their health status and age, ensuring they represent typical individuals of the breed. Importantly, the selected animals were unrelated, minimizing the potential influence of genetic similarities on the observed differences between tissue samples. The experimental procedures and protocol of this study were approved by the Animal Ethics Review Committee of Xinjiang Agricultural University (approval No. 2023004, approval Dade: 15 April 2023). After euthanasia, fresh skeletal muscle tissues were collected from two distinct anatomical locations: the longissimus dorsi (LD) and biceps femoris (BF) muscles. Each muscle type was sampled in triplicate (Figure 1). The samples were immediately snap-frozen in liquid nitrogen and transported on dry ice to the sequencing service provider for further processing.

### 2.2. Single-Cell Suspension Preparation, Single-Cell QC, Doublet Control, and Intronic-Bias Assessment

Frozen muscle tissues were thawed on ice and minced into fine pieces. A two-step enzymatic digestion protocol was applied. First, the tissue was digested in 2 mg/mL Collagenase II (Sigma-Aldrich, St. Louis, MO, USA) at 37 °C for 45 min. After centrifugation and washing, tissues were further digested with 0.25% Trypsin-EDTA (Gibco, Grand Island, NY, USA) for 5–10 min, followed by neutralization with 10% FBS. The resulting cell suspensions were filtered through a 40 µm cell strainer, washed twice with DPBS (0.04% BSA + 0.5 mg/mL DNase I), and resuspended in 1640/DMEM with 5% FBS.

Each single-cell suspension was evaluated prior to library construction. The acceptance criteria were: cell viability >80% by AO/PI staining or >75% by trypan blue exclusion, cell concentration between 700 and 1200 cells/µL, average diameter between 5 and 30 µm, and total viable cell count exceeding 1 × 10^5^. Samples meeting these criteria were selected for downstream processing.

The libraries were prepared and sequenced by a certified sequencing provider. The quality of sequencing was assessed using standard metrics, including raw reads, raw bases, and quality scores (Q20, Q30). The Q20 and Q30 values indicate high-quality sequencing data, with over 98% of bases having accurate calls at Q20 and over 95% base accuracy at Q30. The GC content across samples was consistent, ranging from 39.55% to 40.36%. The sequencing provider’s standard protocols were followed for quality control, and we have included the summary QC parameters in the revised manuscript (Table S1).

### 2.3. Single-Cell RNA Sequencing

High-quality single-cell suspensions were loaded onto the Chromium Controller (10x Genomics, Pleasanton, CA, USA) using the Single Cell 3′ Reagent Kit v3. Gel beads-in-emulsion (GEMs) were generated following the manufacturer’s protocol. Reverse transcription and cDNA amplification were performed in a T100 Thermal Cycler (Bio-Rad, Hercules, CA, USA). After library construction, the libraries were sequenced on an Illumina NovaSeq 6000 platform using the PE150 strategy (paired-end 150 bp reads). Approximately 50,000–100,000 read pairs per cell were generated, with a target sequencing depth of 15–30 million reads per sample.

### 2.4. Read Processing, Mapping, and Primary Analysis

Raw BCL files were demultiplexed using Cell Ranger mkfastq (v6.0.1), and FASTQ files were aligned to the EquCab3.0 reference genome using Cell Ranger count. The pipeline performed barcode processing, UMI counting, and generated a gene-barcode matrix for each sample. Read1 consisted of a 28 bp segment encoding cell barcodes and UMIs, while Read2 (91–100 bp) contained transcript sequence data.

The Cell Ranger alignment process employed STAR for read mapping and utilized GTF annotations to distinguish exonic, intronic, and intergenic reads. Cell calling was based on a refined algorithm that accounts for low RNA-content cells and background correction using empty droplets. Barcodes were ranked by UMI count, and cells were filtered using a cutoff defined by the 99th percentile of top-ranked barcodes. The multiplet rate was estimated by comparing UMI distributions across species using a likelihood-based model.

### 2.5. Quality Control and Filtering

The initial gene expression matrix was filtered using Seurat (v4.3.0) [12]. Cells with fewer than 200 detected genes, more than 6000 genes, or >10% mitochondrial UMI content were excluded. In addition, DoubletFinder was used to identify and remove potential doublets from the dataset, ensuring accurate clustering and reducing artifacts in downstream analyses.

### 2.6. Dimensionality Reduction and Clustering

Filtered count matrices were log-normalized using the “LogNormalize” function in Seurat. Highly variable genes were identified, and data were scaled and subjected to PCA for dimensionality reduction. The top 20 principal components were used to construct a shared nearest neighbor (SNN) graph. Cells were clustered using the Louvain algorithm (resolution = 0.6), and clusters were visualized using UMAP. Cell identities were assigned based on canonical marker gene expression. The top 10 marker genes for each cluster were identified using the Wilcoxon rank-sum test.

### 2.7. Statistical Analysis and Multiple Testing Correction:

To control for multiple testing and reduce the risk of false positives, we applied the Benjamini–Hochberg method for false discovery rate (FDR) correction throughout the analysis. This rigorous method ensures that the FDR is kept at an acceptable level, even with large-scale transcriptomic data. The Benjamini–Hochberg correction was applied to the differential gene expression (DEG) analysis as well as to the KEGG and GO enrichment analyses.

### 2.8. Differentially Expressed Gene (DEG) and Functional Enrichment Analysis

DEG analysis was performed to compare gene expression profiles across different cell clusters using Seurat. To explore the biological functions associated with the differentially expressed genes, we conducted Gene Ontology (GO) and KEGG pathway enrichment analyses using the clusterProfiler package in R [13]. For visualizing the results, we used various R (v 4.4.2) packages, including ggplot2 for generating bar charts, dot plots, and enrichment plots for visualizing heatmaps of enriched pathways and gene clusters [14].

For KEGG pathway enrichment, the top 20 significantly enriched pathways (adjusted *p*-value < 0.05) were visualized using dot plots and bar charts. GO enrichment analysis included terms in biological process (BP), molecular function (MF), and cellular component (CC) categories. Pathways with adjusted *p*-values (Benjamini–Hochberg correction) < 0.05 were considered significantly enriched, and only those involving at least three DEGs and an enrichment factor >1.5 were retained for further analysis.

## 3. Results

### 3.1. Single-Cell RNA-Seq Data Quality and Overall Transcriptomic Output

We generated single-cell transcriptomes from the longissimus dorsi (LD) and biceps femoris (BF) muscles of six tissue samples, with three biological replicates per muscle group. A total of 2.72 billion raw reads were generated from the six libraries, with each sample contributing between 392 million and 578 million reads. The Q30 percentage for all libraries exceeded 95.1%, and GC content ranged from 39.55% to 40.36% (Supplementary Table S1), confirming the high sequencing quality across all samples.

Following Cell Ranger processing and quality filtering, a total of 66,008 high-quality cells were identified, with 36,524 cells from the LD samples and 29,484 cells from the BF samples. The estimated number of cells per sample ranged from 8317 (BF_2) to 14,198 (LD_2), with the mean reads per cell ranging from 31,453 (LD_2) to 54,334 (BF_2). Median genes detected per cell ranged from 948 (LD_2) to 1304 (LD_1), reflecting varying levels of transcript complexity across different muscle groups (Supplementary Table S2).

Sequencing saturation across the samples was generally high, with values ranging from 79.1% (LD_2) to 87.5% (BF_1), indicating that transcript coverage was sufficient for downstream analysis. The reads mapped to the genome ranged from 96.4% to 97.1%, with over 93.9% of reads confidently mapped to the genome. Most of the reads were mapped to intronic regions (~64%), consistent with expectations for single-nucleus or partially fragmented cytoplasmic transcripts in muscle tissue. Notably, the transcriptomic mapping rates ranged from 36.6% (BF_2) to 60.1% (BF_3), reflecting variation in RNA content and complexity among samples (Supplementary Table S2).

We also examined the reads mapped confidently to intergenic, intronic, and exonic regions. The majority of the mapped reads were located in the intronic regions (ranging from 63.7% to 66.3%), which suggests the presence of RNA from different transcriptional regions of the genome, including some fragmented or unspliced RNA. The percentage of reads mapped to exonic regions was consistent across all samples (~9%), while the percentage of transcriptomic reads ranged from 36.6% (BF_2) to 60.1% (BF_3), with the BF samples showing greater variability in transcript complexity.

Furthermore, the fraction of reads within cells ranged from 86.1% to 92.9%, indicating that the majority of the reads were derived from viable cells. The total genes detected per sample ranged from 19,989 to 21,305 genes, indicating broad transcriptional coverage across the samples. The median UMI counts per cell ranged from 1815 (LD_2) to 2929 (BF_3), which reflects the transcript abundance in each sample.

These sequencing data demonstrated high quality across all samples, with adequate coverage, mapping, and cell viability. Subtle differences in gene counts per cell and transcriptomic complexity between LD and BF muscle groups reflect underlying biological variations, which may be attributed to the different functional demands of these two muscle types.

### 3.2. Cell Type Identification and Clustering of LD and BF Muscle Samples

**Figure 2:**
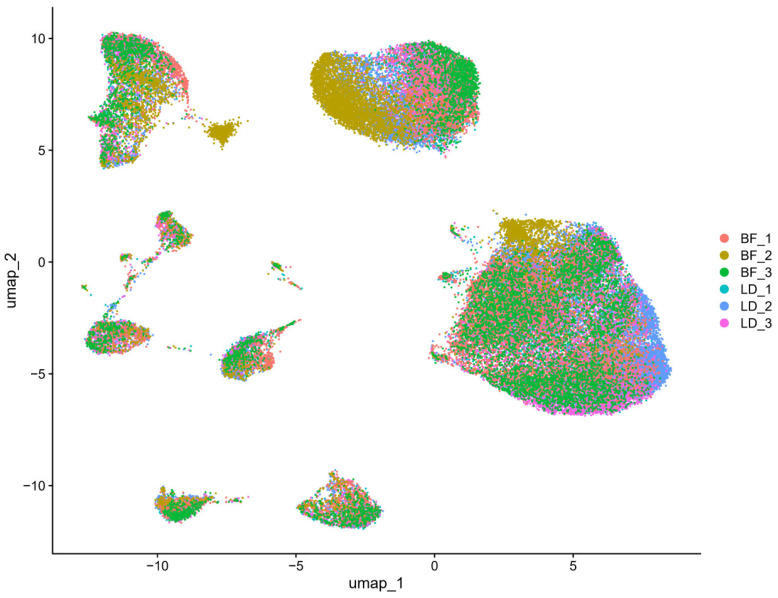
UMAP plot of combined LD and BF muscle samples. The plot shows the clustering of cells from six muscle samples (three from each muscle type, longissimus dorsi (LD) and biceps femoris (BF)). Each color represents a different sample (BF_1, BF_2, BF_3, LD_1, LD_2, LD_3), highlighting the distinct cell distributions across the samples.

Unsupervised clustering of single-cell RNA sequencing (scRNA-seq) data revealed distinct cellular populations in both the LD and BF muscles. After data normalization, scaling, and principal component analysis (PCA), the cellular composition of the muscle samples was visualized using Uniform Manifold Approximation and Projection (UMAP) (Figure 2). Subsequent identification of cell types was performed based on the expression of canonical marker genes. Cells from the LD and BF muscle groups showed clear separation, with distinct cluster patterns observed for each muscle type. The primary cell types identified include myogenic cells (e.g., satellite cells, myoblasts), fibroblasts, endothelial cells, immune cells, and adipocytes. The LD muscle primarily contained myogenic cells, reflecting its role in postural maintenance and stability. In contrast, the BF muscle exhibited a higher proportion of endothelial and stromal cells, which are crucial for muscle contraction and vascularization in the hindlimb.

The distribution of cell types between the two muscle groups was further explored. The LD muscle displayed a higher number of myogenic cells, consistent with its specialized function in posture and back extension. Meanwhile, the BF muscle exhibited a more diverse cell composition, reflecting its involvement in dynamic movements such as limb propulsion. This analysis indicates the functional specialization of the muscle groups and underscores their differences in cellular makeup.

### 3.3. Differential Gene Expression (DEG) Analysis Between LD and BF Muscle

**Figure 3:**
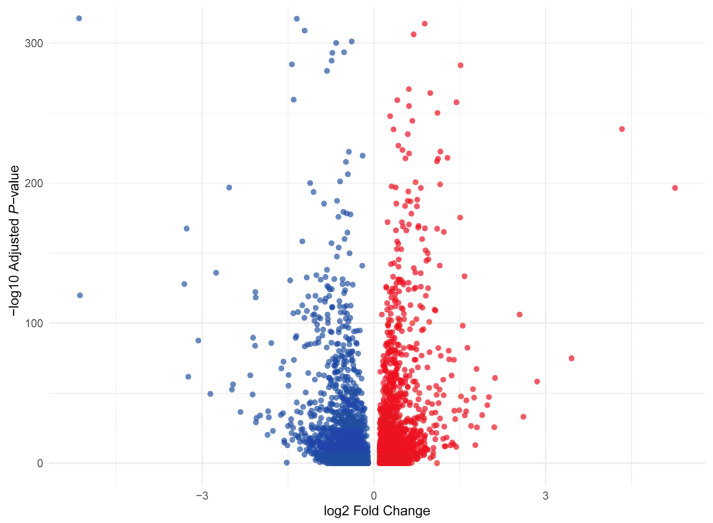
Volcano Plot of Differential Gene Expression Between LD and BF Muscle.

Differential gene expression analysis revealed 6910 differentially expressed genes (DEGs) between longissimus dorsi (LD) and biceps femoris (BF) muscle cells (Figure 3 and Supplementary Table S3). Among these, 1545 genes were upregulated in LD, while 1734 genes were downregulated in LD compared to BF (Supplementary Tables S4 and S5). It is important to note that these DEGs were identified based on a comparison across all cells from the LD and BF muscle types, rather than being analyzed per individual cluster. The differences in gene expression between the two muscle types reflect the overall cellular composition, but they do not account for the specific variation between cell types within each cluster. Future analyses may focus on per-cluster DEGs to explore more specific gene expression patterns within distinct cell populations. The most significantly upregulated genes in LD compared to BF include MYL1, a key player in muscle contraction, which was upregulated with a log2FC of 1.18, highlighting enhanced muscle contraction mechanisms in LD. TXNIP, involved in oxidative stress, showed a log2FC of 0.47, indicating a stronger oxidative stress response in LD muscle cells. LDHA, crucial for anaerobic metabolism, was upregulated with a log2FC of 1.19, suggesting LD muscle cells’ reliance on anaerobic glycolysis. PFKFB3, regulating glycolysis, had a log2FC of 1.25, further supporting enhanced glycolytic activity in LD. PEBP4, involved in cell signaling pathways, was upregulated with a log2FC of 1.01, reflecting the importance of cellular communication in muscle adaptation during exercise or stress. Additional genes upregulated in LD include MYLK2 (log2FC = 0.89), involved in smooth muscle contraction, suggesting the importance of contractile proteins; EGF (log2FC = 1.83), associated with cell growth and development, indicating cellular growth activity in LD muscle; MYOM2 (log2FC = 0.69), which is involved in muscle protein synthesis, further supporting enhanced muscle function in LD; GADL1 (log2FC = 1.32), related to metabolism and signaling, showing increased metabolic activity in LD; and PGM1 (log2FC = 1.34), involved in energy metabolism, suggesting efficient energy production in LD muscle cells.

The most downregulated genes in LD compared to BF include LEP, a hormone regulating energy balance and fat storage, which was downregulated with a log2FC of −0.56, indicating reduced fat storage in LD. RBP4, involved in vitamin A transport and insulin resistance, showed a downregulation with a log2FC of −0.71. PLIN4, which plays a role in lipid droplet formation, was downregulated with a log2FC of −0.57, suggesting reduced lipid storage in LD muscle cells compared to BF. INSIG1 (log2FC = −0.59) and ADIPOQ (log2FC = −0.61), both involved in insulin signaling and adipogenesis, were downregulated, reflecting altered metabolic regulation in LD. Additional genes downregulated in LD include FABP5 (log2FC = −0.85), involved in lipid binding and transport, supporting the reduced reliance on lipid metabolism; AGPAT2 (log2FC = −0.75), involved in glycerolipid biosynthesis, reinforcing the idea that LD muscle cells utilize alternative energy sources; SLC16A7 (log2FC = −1.78), related to monocarboxylate transport, downregulated, further supporting the shift in metabolic processes; SLC25A21 (log2FC = −0.67), a mitochondrial transporter, suggesting changes in mitochondrial function in LD; and MT-CO2 (log2FC = −0.51), part of the mitochondrial respiratory chain, showing reduced mitochondrial activity in LD.

### 3.4. Functional Analysis Indicates Activation of Muscle-Specific Pathways in LD

**Figure 4:**
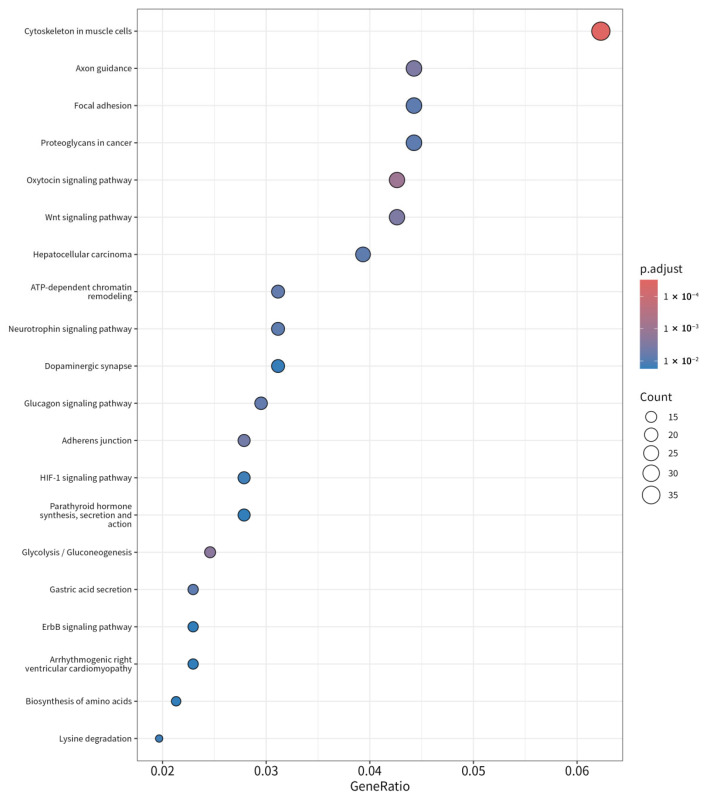
KEGG Pathway Enrichment Analysis of Upregulated Genes in LD Compared to BF Muscle.

Functional analysis of the differential gene expression between LD and BF was performed using KEGG pathway enrichment analysis (Supplementary Table S6). The top 5 activated and suppressed KEGG pathways in LD compared to BF are presented in Figure 4. The analysis revealed a suppression of pathways associated with lipid metabolism, including cholesterol metabolism, fatty acid metabolism, and PPAR signaling. These results suggest that LD muscle cells may rely less on lipid metabolism compared to BF muscle cells, which are more associated with energy storage and lipid utilization.

In contrast, the KEGG analysis highlighted the activation of several key pathways in LD, including cardiac muscle contraction, calcium signaling, and MAPK signaling. These pathways are critical for muscle contraction, calcium homeostasis, and cellular responses to external stimuli, all of which are essential for muscle function in LD (Figure 4). The activation of these pathways suggests that LD muscle cells are highly active in terms of energy production, oxidative stress handling, and calcium regulation, which are essential for muscle contraction and endurance. Furthermore, neuro-related signaling pathways, such as those involved in amyotrophic lateral sclerosis (ALS), Alzheimer’s, and Parkinson’s diseases, were activated in LD, indicating that LD muscle cells have enhanced signaling mechanisms for maintaining muscle activity and communication with the nervous system.

Further analysis revealed the activation of pathways crucial for muscle development and function, such as the calcium signaling pathway (involved in maintaining cellular calcium levels critical for muscle contraction), and cardiac muscle contraction pathway, which shares similar functional characteristics with skeletal muscle contraction. The MAPK signaling pathway, involved in cellular response to stress and injury, was also significantly activated, supporting the adaptive response of muscle cells in LD to external stimuli. Additionally, pathways associated with neurodegenerative diseases like amyotrophic lateral sclerosis (ALS) were activated, suggesting a high degree of coordination between muscle cells and the nervous system, essential for muscle contraction and endurance.

**Figure 5:**
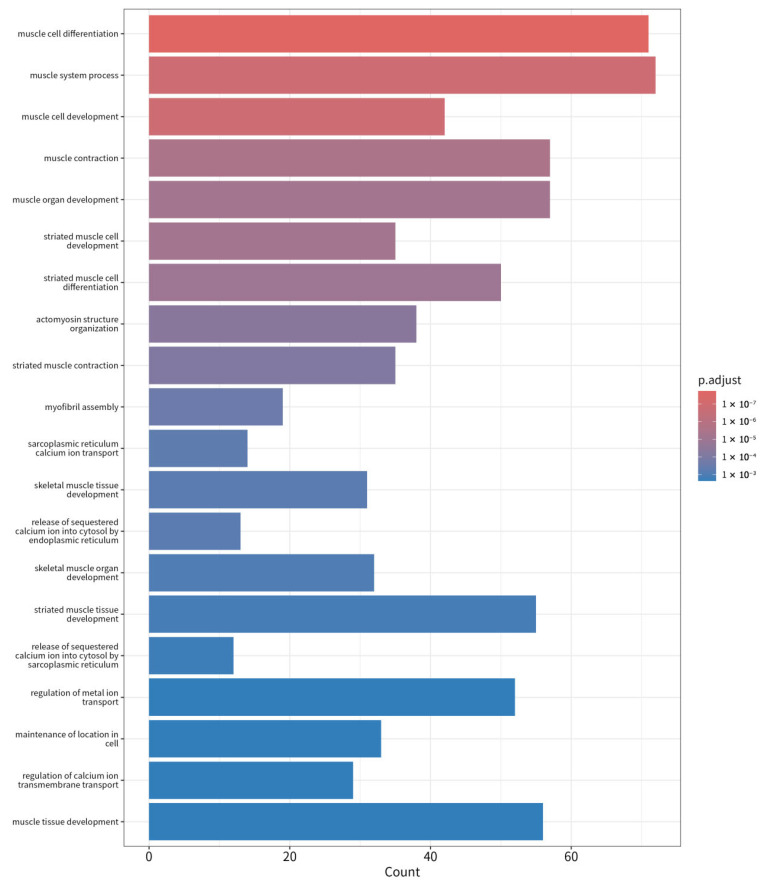
GO Enrichment Analysis of Upregulated Genes in LD Compared to BF Muscle.

Additionally, Gene Ontology (GO) term analysis confirmed that the upregulated genes in LD were significantly involved in muscle structure development (Figure 5), actin cytoskeleton organization, and muscle contraction, which aligns with the muscle-specific pathways identified in KEGG analysis (Supplementary Table S7). Key genes such as MYOD1, ATP2A1, and ACTA1, which play pivotal roles in muscle development and contraction, were significantly upregulated in LD, supporting their involvement in muscle function. These pathways emphasize the role of LD muscle cells in maintaining muscle strength, energy production, and responsiveness to external stress.

## 4. Discussion

In this study, we utilized scRNA-seq to explore the transcriptomic differences between the LD and BF muscles in Kazakh horses. Our results provide novel insights into how these muscles, which serve distinct functional roles, differ at the cellular and molecular levels. Kazakh horses are known for their endurance and adaptability to harsh environments, and this functional differentiation between muscle groups supports their specialized roles in postural support (LD) and dynamic movements (BF).

The cellular composition of the two muscles revealed clear functional specialization. The LD muscle, responsible for postural maintenance and endurance, showed a predominance of myogenic cells, including satellite cells and myoblasts [15]. This cellular composition aligns with the muscle’s role in sustaining long-term contractions and its adaptation to endurance activities. The BF muscle, on the other hand, exhibited a higher proportion of endothelial and stromal cells, which are crucial for muscle contraction and vascularization during dynamic, forceful movements [16,17]. These differences in cellular composition are consistent with the specialized roles of these muscles, as described in previous studies [18,19,20]. Previous studies demonstrated that muscles involved in endurance activities exhibit a higher proportion of Type I fibers (slow-twitch fibers), while muscles involved in rapid, high-intensity movements predominantly consist of Type II fibers (fast-twitch fibers) [21].

Gene expression analysis further emphasized the metabolic and functional specialization of the LD and BF muscles. In the LD muscle, genes associated with muscle contraction, anaerobic metabolism, and oxidative stress response were significantly upregulated. MYL1, a gene encoding for myosin light chain, was notably upregulated, reflecting its role in muscle contraction and endurance [6,22]. Similar findings have been reported in other equine studies, where MYL1 is associated with slow-twitch fibers, which are essential for prolonged activity [23]. The upregulation of LDHA, a key enzyme in anaerobic glycolysis, indicates that the LD muscle relies heavily on glycolysis for energy production, a hallmark of endurance muscle fibers [24]. TXNIP, which regulates oxidative stress, was found to be upregulated in LD muscle, suggesting that these cells may be under greater oxidative stress, a characteristic commonly observed in muscles engaged in long-duration, endurance-type activity. While TXNIP has been well documented in human and mouse studies as a key regulator of oxidative stress response pathways, its exact role in equine skeletal muscle remains speculative [25,26,27]. Given the upregulation of TXNIP in LD muscle, we hypothesize that LD muscle cells may be exposed to higher levels of oxidative stress, possibly due to prolonged muscle contraction during endurance activities. However, we acknowledge that further experimental validation (e.g., qPCR or protein-level confirmation) is necessary to establish a direct functional link between TXNIP and oxidative stress in Kazakh horses [27]. This finding is in line with previous research that identified increased oxidative stress in endurance muscles, contributing to their adaptation to prolonged contraction.

In contrast, the BF muscle exhibited downregulation of genes involved in glycolysis and lipid metabolism, such as LEP and FABP5, which regulate energy balance and fat storage [28,29]. The downregulation of these genes suggests a metabolic shift in BF muscle cells, favoring glycolysis and rapid energy production rather than lipid storage. This is consistent with the muscle’s role in high-intensity, short-duration activities, where rapid energy mobilization is critical. The downregulation of FABP5 also suggests a reduced reliance on lipids for fuel, which aligns with findings in fast-twitch muscle fibers that primarily use glucose for energy during intense exercise [30,31]. The downregulation of INSIG1 and ADIPOQ, genes associated with insulin signaling and adipogenesis, also supports the concept that BF muscle cells favor glycolytic pathways [32,33]. These genes are typically involved in lipid storage and energy regulation, suggesting that BF muscle cells shift away from lipid metabolism, likely to ensure rapid mobilization of energy during short, intense bursts of activity [9,34].

In terms of KEGG pathway enrichment, the analysis revealed that LD muscle cells were enriched in pathways related to muscle contraction, calcium signaling, and MAPK signaling [35]. These pathways are crucial for maintaining muscle function during sustained contraction, as they regulate calcium homeostasis and the cellular response to external stimuli. The activation of MAPK signaling is particularly relevant in the context of endurance, as this pathway is involved in muscle adaptation to stress and injury [36,37,38,39]. Conversely, BF muscle cells were enriched in glycolytic and energy metabolism pathways, suggesting that the muscle cells prioritize rapid energy production to support high-intensity contractions. This is consistent with the functional requirements of BF, which is specialized for explosive movements [40].

Interestingly, both muscle groups exhibited differences in their immune-related gene expression. BF muscle showed enhanced immune-related pathways, which may reflect its role in maintaining muscle integrity during intense physical activity. In contrast, LD muscle exhibited lower immune activity, which is typically observed in muscles involved in endurance, where immune responses are less pronounced due to the less strenuous nature of the activity [41,42]. These immune-related differences further highlight the functional specialization of each muscle type.

In our analysis, we observed enrichment in pathways related to neurodegenerative diseases such as ALS (Amyotrophic Lateral Sclerosis) in LD muscle. While this finding is intriguing, the relationship between these pathways and skeletal muscle function is not fully understood. ALS is primarily a neurodegenerative disorder that affects motor neurons; however, skeletal muscle involvement in ALS, including the presence of oxidative stress, inflammation, and apoptosis, is well-documented in other species [43,44]. One possible hypothesis is that the upregulation of neurodegenerative disease-related pathways in LD muscle could reflect a cellular stress response that is similar to what is seen in ALS. Skeletal muscles in long-duration, endurance activities, like those in Kazakh horses, could experience chronic oxidative stress and cellular damage, which might mimic some aspects of neurodegenerative disease pathways. For example, oxidative stress is a well-known trigger for apoptotic signaling and neuroinflammation, both of which are common in ALS.

This study provides a detailed view of the transcriptional landscape of Kazakh horse muscle tissues and underscores the complex biological mechanisms that drive muscle specialization. By examining the differential expression of key genes involved in metabolism, muscle contraction, and immune regulation, we have gained insights into the molecular adaptations of these muscles to their specific functional demands. These findings have important implications for understanding how different muscle types in Kazakh horses, and other equine breeds, adapt to their specialized roles in endurance and dynamic movements. Furthermore, the study of muscle tissue in Kazakh horses is critical for improving breeding programs aimed at enhancing specific traits related to muscle function. Our findings suggest that LD muscle is optimized for endurance and sustained activity, while BF muscle is specialized for rapid, explosive movements. This knowledge can inform breeding decisions that focus on improving performance in different equine disciplines. Future studies should aim to validate these findings through functional experiments and explore the potential for manipulating key genes, such as MYL1, LDHA, and TXNIP, to enhance muscle function in horses. Additionally, the integration of other factors, such as breed, nutrition, and exercise regimes, into the analysis of muscle tissue could provide further insights into the mechanisms driving muscle performance and adaptation in Kazakh horses.

We acknowledge several limitations in our study, which should be considered when interpreting the results. Firstly, the small sample size (n = 3) limits our ability to fully account for biological variability and to generalize the findings. In particular, we were unable to perform additional statistical analyses, such as the chi-square test, to assess the significance of differences in cell-type proportions between the longissimus dorsi (LD) and biceps femoris (BF) muscles. While we observed a predominance of myogenic cells in LD and endothelial/stromal cells in BF, we could not conclusively determine whether these differences reflect true biological variations or if they are influenced by dissociation biases, such as the potential difficulty in isolating myogenic cells from BF muscle. Future studies with larger sample sizes and improved tissue dissociation techniques will be necessary to address these uncertainties and validate the findings. Additionally, biological variability due to factors such as sex, age, environmental conditions, and farm management practices (e.g., diet, housing, and handling) could influence gene expression in muscle tissues. Although we aimed to minimize potential confounders by selecting healthy, age-matched male horses, the small sample size limits our ability to fully control for these variables. Further studies with larger and more controlled samples are recommended to better generalize these findings. Given the limitations of our sample size, we acknowledge that the robustness of our results should be interpreted with caution. Although we did not perform power calculations for this pilot study, we suggest that future work should include power analysis to determine the necessary sample size for detecting statistically significant differentially expressed genes (DEGs) with sufficient power. Sensitivity analyses could also be conducted to assess whether the identified DEGs remain consistent across varying conditions and larger cohorts. To strengthen the findings of this study, we plan to validate key DEGs, such as MYL1 and LDHA, using quantitative PCR (qPCR) on independent samples. Furthermore, additional data from a larger number of horses, including horses from different farms or exposed to varying environmental conditions, will provide a more comprehensive understanding of muscle-specific gene expression and the impact of genetic and environmental factors on muscle performance in Kazakh horses.

## 5. Conclusions

This study provides valuable insights into the transcriptomic differences between the LD and BF muscles in Kazakh horses, revealing their functional specialization at the molecular level. The LD muscle, optimized for endurance, showed upregulation of genes involved in anaerobic metabolism and muscle contraction, while the BF muscle, specialized for explosive movements, displayed a shift toward lipid metabolism and rapid energy production. These findings highlight the metabolic and functional divergence between the two muscle types, with distinct pathway enrichments further supporting their specialized roles.

The results from this study offer a deeper understanding of muscle biology in Kazakh horses and provide potential directions for breeding programs focused on optimizing muscle performance and health in equines. Specifically, the identified gene expression profiles and metabolic pathways could be integrated into breeding strategies to enhance traits such as endurance, muscle strength, and disease resistance. This research not only benefits Kazakh horses but also has broader implications for other equine breeds, as the metabolic pathways identified are conserved across species. Breeding programs that target these pathways could improve overall equine health and performance, with particular applications in competitive and working horses.

## Data Availability

The data presented in this study are available on request from the corresponding author (J.W.).

## Supplementary Materials

The following supporting information can be downloaded at https://www.mdpi.com/article/10.3390/ani15192778/s1, Supplementary Table S1: Raw Sequencing Data Quality Metrics for LD and BF Samples; Supplementary Table S2: Summary of Sequencing and Mapping Metrics for BF and LD Samples; Supplementary Table S3: Summary of DEGs between BF and LD Samples; Supplementary Table S4: Upregulated of DEGs between BF and LD Samples; Supplementary Table S5: Downregulated of DEGs between BF and LD Samples; Supplementary Table S6: KEGG Pathway Enrichment Analysis for Upregulated Genes; Supplementary Table S7: Upregulated Gene Ontology Enrichment Analysis for Biological Processes.
